# Artificial intelligence-based decision support in simulated free flap *Re*-exploration for head and neck reconstruction. A case-based comparative study

**DOI:** 10.1016/j.jpra.2026.05.010

**Published:** 2026-05-16

**Authors:** Sebastian Holm, Juan E Berner, Ann-Charlott Docherty Skogh, Dhafir Hanoon, Mattias Lidén, Gabriel Badida, Fredrik Landström, Reza Tabrisi

**Affiliations:** a Department of Reconstructive Plastic Surgery, Karolinska University hospital, Stockholm, Sweden; b Faculty of Medicine and Health, Örebro University, Örebro, Sweden; c Division of Plastic and Reconstructive Surgery, Department of Surgery, University of Texas at San Antonio, San Antonio, Texas, United States; d Department of Molecular Medicine and Surgery, Karolinska Institutet, Stockholm, Sweden; e Department of Plastic and Reconstructive Surgery, Örebro University Hospital, Örebro, Sweden; f Department of Plastic Surgery, Sahlgrenska University Hospital, Gothenburg, Region Västra Götaland, Sweden; g Department of Plastic Surgery, Institute of Clinical Sciences, Sahlgrenska Academy, University of Gothenburg, Gothenburg, Sweden; h Department of Plastic and Aesthetic Surgery, Olomouc University Hospital, Olomouc, Czech Republic; i Faculty of Medicine and Dentistry, Palacky University Olomouc, Olomouc, Czech Republic; j Department of Otorhinolaryngology, Örebro University Hospital, Örebro, Sweden; k Migraine Surgery Centre Scandinavia, Gothenburg, Sweden

**Keywords:** Free flap re-exploration, Head and neck reconstruction, Microsurgery, Large language models, Clinical decision support, Artificial intelligence

## Abstract

Free flap compromise after head and neck reconstruction requires rapid recognition and structured escalation. Large language models have shown potential for text-based clinical support, but their alignment with microsurgical decision-making in flap compromise remains unclear. This simulated case study evaluated three large language models, ChatGPT, Gemini, and Copilot, using 30 standardized head and neck free flap compromise scenarios, including 15 intraoperative and 15 postoperative cases. Each model received identical prompts requesting the suspected diagnosis, likely mechanism of compromise, and recommended management steps. Four consultant microsurgeons independently rated each response using 5-point Likert scales across clinical accuracy, management recommendations, assessment correctness, usefulness of explanation, and overall confidence. Potential for harm was rated as yes or no Ratings were aggregated to case level. Between-model comparisons were performed separately for intraoperative and postoperative scenarios using Friedman tests with Bonferroni-adjusted post hoc Wilcoxon signed-rank tests, while potential for harm was compared using chi-square tests.

Clinical accuracy ratings were high across all models in both intraoperative and postoperative scenarios, with no significant between-model differences. In intraoperative cases, usefulness of explanation differed significantly between models, with lower ratings for Copilot than Gemini. In postoperative cases, Gemini received significantly higher ratings for management recommendations and usefulness of explanation than ChatGPT and Copilot, and higher overall confidence than Copilot. Potentially harmful recommendations were uncommon, but differed between models in postoperative scenarios. Interrater agreement was generally higher in intraoperative than postoperative cases.

Large language models showed high alignment with expert ratings for recognition of flap compromise in standardized simulated scenarios. However, differences emerged in management planning, explanatory clarity, and confidence, particularly in postoperative cases. These findings support cautious interpretation and do not support independent clinical use in microsurgical care.

## Introduction

Microsurgical free flap reconstruction is an established approach for reconstructing complex defects in head and neck surgery, with reported success rates ranging from 90 to 95%.^1^^,^^2^ Despite this, 5–25% of flaps require re-exploration for suspected post-operative vascular compromise.^3^, ^4^, ^5^ Venous thrombosis is the predominant cause of flap loss in this group (35%), while arterial and mechanical events such as kinking or compression account for the remainder.^1^^,^^6^ Most emergency re-exploration occur within the first 48 h after reconstruction, with early re-exploration resulting in higher salvage rates.^3^^,^^5^^,^^7^ Inadequate recognition of early signs or hesitation lower the chance of salvage and expose patients to flap loss, repeat reconstruction and prolonged hospital stay.^1^^,^^7^

The interest for application of artificial intelligence (AI) in plastic and reconstructive surgery is increasing.^8^^,^^9^ Some recent studies describe applications of machine learning (ML) and large language models (LLM) in preoperative planning, for intraoperative decision support and postoperative monitoring in microsurgical reconstruction.^10^, ^11^, ^12^, ^13^, ^14^, ^15^, ^16^, ^17^ LLM process free text and produce structured clinical reasoning which offers a potential decision support tool for complex scenarios such as flap compromise.^16^ LLMs support in plastic surgery education has also been proposed to be incorporated in education for trainees.^18^

To our knowledge, no prior study has evaluated LLM performance in standardized free flap re-exploration decision for head and neck reconstruction. Free flap compromise is a high-stakes event with a narrow salvage window, making timely recognition and structured escalation essential.

The aim of this simulated case study was to evaluate how responses from three LLMs aligned with consultant microsurgeon judgement in recognizing flap compromise, proposing management steps and supporting escalation in intraoperative and postoperative scenarios. The study was designed to assess alignment with expert reasoning in a controlled scenario-based setting, not to establish clinical effectiveness in real patient care.

## Methods

### Study design

This is a prospective simulated case study with a multi-institutional, international expert panel. The study evaluated three LLMs as text-based reasoning support systems in standardized free flap compromise scenarios after head and neck reconstruction. Thirty simulated case scenarios were used, comprising 15 intraoperative and 15 postoperative cases, and all responses were blindly evaluated by four consultant microsurgeons (DH, ACDS, GB, ML). No real patient data were used. The purpose of this design was to allow controlled comparison of model outputs in a high-risk microsurgical setting without exposing patients to unsafe recommendations.

### Development of simulated cases

Thirty simulated case scenarios were constructed to reflect typical etiologies of free-flap compromise. Fifteen scenarios represented intraoperative events and 15 represented postoperative deterioration. The distribution reflected clinical routine on venous compromise, arterial insufficiency, inflow or outflow obstruction, pedicle mechanical issues, systemic factors and rare late complications. Each case scenario followed the same structured template. This included flap type, indication, timing, visual description, Doppler findings and key examination features. The postoperative cases were designed as static clinical snapshots rather than evolving real-time course. (Cases can be found in Supplement Table 1).

**Table 1:** Descriptive statistics for intraoperative and postoperative simulated cases (n = 30).

Intraoperative simulated cases			
Domain	Model A mean (SD), Min-Max	Model B mean (SD), Min-Max	Model C mean (SD), Min-Max
Clinical accuracy (CA)	4.67 (0.44), 4.25–5.00	4.47 (1.03), 4.25–5.00	4.85 (0.26), 4.25–5.00
Management recommendations (MR)	4.53 (0.46), 4.00–4.75	4.47 (0.96), 4.25–5.00	4.28 (0.38), 3.25–4.75
Assessment correctness (AC)	4.73 (0.23), 4.25–5.00	4.58 (0.67), 4.25–5.00	4.65 (0.29), 4.00–5.00
Usefulness of explanation (UE)	4.68 (0.31), 4.00–5.00	4.58 (0.86), 4.75–5.00	4.28 (0.33), 3.25–4.75
Overall confidence (OC)	4.65 (0.27), 4.00–5.00	4.58 (0.77), 4.25–5.00	4.38 (0.43), 3.50–5.00

Postoperative simulated cases			
Domain	Model A mean (SD), Min-Max	Model B mean (SD), Min-Max	Model C mean (SD), Min-Max

Clinical accuracy (CA)	4.75 (0.27), 4.25–5.00	4.92 (0.12), 4.75–5.00	4.80 (0.25), 4.25–5.00
Management recommendations (MR)	4.40 (0.26), 4.00–4.75	4.87 (0.23), 4.25–5.00	4.17 (0.53), 3.25–4.75
Assessment correctness (AC)	4.73 (0.24), 4.25–5.00	4.85 (0.25), 4.25–5.00	4.60 (0.39), 4.00–5.00
Usefulness of explanation (UE)	4.53 (0.34), 4.00–5.00	4.93 (0.11), 4.75–5.00	4.15 (0.52), 3.25–4.75
Overall confidence (OC)	4.62 (0.33), 4.00–5.00	4.90 (0.21), 4.25–5.00	4.38 (0.50), 3.50–5.00

### LLM and standardized prompting

Three large language models were tested using the public web-based interfaces available during the study period: ChatGPT, GPT-4o (OpenAI, San Francisco, CA, USA), Gemini Advanced, Gemini 1.5 Pro (Google DeepMind, Mountain View, CA, USA), and Microsoft Copilot, GPT-4-based web interface (Microsoft, Redmond, WA, USA). All model responses were generated 15th of December 2025. These models were blinded for the expert panel as Model A (ChatGPT), Model B (Gemini) and Model C (Copilot). Each model received identical case scenarios in randomized order. A fixed prompt structure ensured consistency across the models and the cases. Prompts requested three outputs: the suspected diagnosis, the likely the mechanism of compromise and the recommendation management steps. (The prompts used can be read in detail in Supplement Table 2).

**Table 2:** Intraoperative clinical accuracy (CA), management recommendations (MR), assessment correctness (AC), usefulness of explanation (UE) and overall confidence (OC) with Friedman test and post hoc pairwise comparisons.

Intraoperative clinical accuracy (CA):			

Friedman test summary			
Chi-square	Degree of freedom	p-value	
0.76	2	0.68	
Post hoc pairwise comparisons (Wilcoxon signed-rank test with Bonferroni correction			
Comparison	Test statistics	p-value	Adjusted p-value
Model A vs B	−0.13	0.71	1.00
Model A vs C	−0.26	0.46	1.00
Model B vs C	−0.13	0.71	1.00

Intraoperative management recommendations (MR):			

Friedman test summary			
Chi-square	Degree of freedom	p-value	
5.67	2	0.059	
Post hoc pairwise comparisons (Wilcoxon signed-rank test with Bonferroni correction			
Comparison	Test statistics	p-value	Adjusted p-value
Model A vs B	−0.20	0.58	1.00
Model A vs C	0.60	0.10	0.30
Model B vs C	0.80	0.02	0.08

Intraoperative usefulness of explanation (UE):			

Friedman test summary			
Chi-square	Degree of freedom	p-value	
9.59	2	0.008	
Post hoc pairwise comparisons (Wilcoxon signed-rank test with Bonferroni correction			
Comparison	Test statistics	p-value	Adjusted p-value
Model A vs B	−0.26	0.46	1.00
Model A vs C	0.76	0.03	0.10
Model B vs C	1.03	0.005	0.014

Intraoperative overall confidence (OC):			

Friedman test summary			
Chi-square	Degree of freedom	p-value	
6.28	2	0.043	
Post hoc pairwise comparisons (Wilcoxon signed-rank test with Bonferroni correction			
Comparison	Test statistics	p-value	Adjusted p-value
Model A vs B	−0.36	0.31	0.94
Model A vs C	0.46	0.20	0.60
Model B vs C	0.83	0.02	0.06

Intraoperative assessment correctness (AC):			

Friedman test summary			
Chi-square	Degree of freedom	p-value	
2.28	2	0.31	
Post hoc pairwise comparisons (Wilcoxon signed-rank test with Bonferroni correction			
Comparison	Test statistics	p-value	Adjusted p-value
Model A vs B	0.00	1.00	1.00
Model A vs C	0.40	0.27	0.82
Model B vs C	0.40	0.27	0.82

### Expert panel evaluation

Four consultant microsurgeons (D.H, M.L, G.B, AC.DS) served as the reference standard from University hospitals in Sweden (Stockholm, Gothenburg and Örebro) and one university hospital in Czech Republic. Each expert received the same full case set. The domains assessed were: Clinical accuracy of diagnosis (CA), appropriate management recommendations (MR), assessment correctness (AC), clarity and usefulness of explanation (UE), potential for harm (PH) and overall confidence (OC) with response. Ratings were collected using a pre-defined Excel sheet with a 5-point Likert scale. Potential for harm (PH) was evaluated as yes or no The panel size was limited and individual rater scoring therefore had a greater influence on aggregated case-level means than would be expected in a larger expert panel. The findings should therefore be interpreted as a focused expert comparison rather than a consensus estimate for the wider microsurgical community.

### Statistical analysis

All statistical analysis were performed using IBM SPSS Statistics (IBM Corp., Armonk, NY, USA). Thirty simulated free flap compromise scenarios were analysed, comprising 15 intraoperative and 15 postoperative cases. For each case, model and domain, rater scores were aggregated to a single case-level value prior to between-model comparison. Descriptive statistics were calculated separately for intraoperative and postoperative cases. Due to evaluation domains being ordinal and the same cases were assessed across all three models, between-model comparisons were performed using Friedman´s test for repeated ordinal measures. Friedman tests and post hoc pairwise comparisons using Wilcoxon signed-ranked tests with Bonferroni correction were done. Potential for harm was analysed descriptively and compared between models using Pearson´s chi-square test. Interrater reliability was assessed separately for each model and scenario type, intraoperative and postoperative. Agreement between raters was quantified using Cohen´s kappa coefficient for each evaluation domain and for potential for harm. Cohen´s kappa coefficient over 0.7 were evaluated as agreement between raters.

## Results

Thirty simulated free flap compromise cases were analysed, including 15 intraoperative and 15 postoperative scenarios. Case-level descriptive statistics are shown in Table 1. Between-model comparisons for intraoperative cases are shown in Table 2, Table 3. Between-model comparisons for postoperative cases are shown in Table 4, Table 5. Interrater reliability results are shown in Table 6.

**Table 3:** Potential for harm (pH) with chi-square tests for differences in potential for harm classification across models in intraoperative simulated cases (n = 15 cases, 45 model evaluations).

Test	Chi-square value	Degrees of freedom	p-value
Pearson Chi-square	0.45	2	0.79
Likelihood ratio	0.48	2	0.78
Linear-by linear association	0.30	1	0.56

**Table 4:** Postoperative clinical accuracy (CA), management recommendations (MR), assessment correctness (AC), usefulness of explanation (UE), overall confidence (OC) with Friedman test and post hoc pairwise comparisons.

Postoperative management recommendations (MR):			
Friedman test summary			
Chi-square	Degree of freedom	p-value	
17.46	2	<0.001	
Post hoc pairwise comparisons (Wilcoxon signed-rank test with Bonferroni correction			
Comparison	Test statistics	p-value	Adjusted p-value
Model A vs B	−1.10	0.003	0.008
Model A vs C	−0.30	0.41	1.00
Model B vs C	−1.40	<0.001	0.0003781

Postoperative assessment correctness (AC):			

Friedman test summary			
Chi-square	Degree of freedom	p-value	
5.15	2	0.076	
Post hoc pairwise comparisons (Wilcoxon signed-rank test with Bonferroni correction			
Comparison	Test statistics	p-value	Adjusted p-value
Model A vs B	−0.43	0.23	0.70
Model A vs C	−0.23	0.52	1.00
Model B vs C	−0.66	0.06	0.20

Postoperative usefulness of explanation (UE):			

Friedman test summary			
Chi-square	Degree of freedom	p-value	
22.84	2	<0.001	
Post hoc pairwise comparisons (Wilcoxon signed-rank test with Bonferroni correction			
Comparison	Test statistics	p-value	Adjusted p-value
Model A vs B	−0.90	0.014	0.041
Model A vs C	−0.80	0.028	0.085
Model B vs C	−1.70	<0.001	0.0000096

Postoperative overall confidence (OC):			

Friedman test summary			
Chi-square	Degree of freedom	p-value	
10.97	2	0.004	
Post hoc pairwise comparisons (Wilcoxon signed-rank test with Bonferroni correction			
Comparison	Test statistics	p-value	Adjusted p-value
Model A vs B	−0.66	0.068	0.20
Model A vs C	−0.36	0.31	0.94
Model B vs C	−1.03	0.005	0.014

**Table 5:** Potential for harm (pH) with chi-square tests for differences in potential for harm classification across models in postoperative simulated cases (n = 15 cases, 45 model evaluations).

Test	Chi-square value	Degrees of freedom	p-value
Pearson Chi-square	8.78	2	0.012
Likelihood ratio	9.59	2	0.008
Linear-by linear association	6.43	1	0.01

**Table 6:** Interrater reliability across all models and intraoperative and postoperative cases.

Model A	CA	MR	AC	UE	OC	pH

Intraoperative	0.523	0.359	0.290	0.285	0.147	0.788
Postoperative	0.186	0.110	0.012	0.248	0.250	>0.8

Model B	CA	MR	AC	UE	OC	pH

Intraoperative	0.937	0.897	0.599	0.895	0.802	0.929
Postoperative	0.522	0.576	0.617	−0.273	0.611	>0.8

Model C	CA	MR	AC	UE	OC	pH

Intraoperative	0.703	0.475	0.469	0.414	0.672	1.00
Postoperative	0.404	0.593	0.600	0.603	0.678	0.616

### Intraoperative results

Fifteen intraoperative simulated cases were analysed. Clinical accuracy did not differ between models (Friedman chi-square = 0.76, df = 2, *p* = 0.68). Management recommendations did not differ significantly between models (chi-square = 5.67, df = 2, *p* = 0.059). Assessment correctness did not differ between models (chi-square = 2.28, df = 2, *p* = 0.31). Usefulness of explanation differed between models (chi-square = 9.59, df = 2, *p* = 0.008), with lower ratings for Copilot compared with Gemini after Bonferroni adjustment (adjusted *p* = 0.014). Overall confidence differed in the Friedman test (chi-square = 6.28, df = 2, *p* = 0.043), but no pairwise comparison remained significant after Bonferroni adjustment. Potential for harm did not differ between models (Pearson chi-square = 0.45, df = 2, *p* = 0.79). (Table 2, Table 3).

### Postoperative results

Fifteen postoperative simulated cases were analysed. Clinical accuracy did not differ between models (Friedman chi-square = 3.60, df = 2, *p* = 0.165). Management recommendations differed between models (chi-square = 17.46, df = 2, *p* < 0.001), with higher ratings for Gemini compared with ChatGPT (adjusted *p* = 0.008) and Copilot (adjusted *p* = 0.0003781). Assessment correctness did not differ between models (chi-square = 5.15, df = 2, *p* = 0.076). Usefulness of explanation differed between models (chi-square = 22.84, df = 2, *p* < 0.001), with higher ratings for Gemini compared with ChatGPT (adjusted *p* = 0.041) and Copilot (adjusted *p* = 0.0000096). Overall confidence differed between models (chi-square = 10.97, df = 2, *p* = 0.004), with higher ratings for Gemini compared with Copilot (adjusted *p* = 0.014). Potential for harm differed between models (Pearson chi-square = 8.78, df = 2, *p* = 0.012). (Table 4, Table 5)

### Interrater reliability

Interrater reliability differed markedly by scenario type, evaluation domain and model (Table 6). Agreement was consistently higher for intraoperative than postoperative cases across all three models. In intraoperative cases, Gemini showed the highest interrater agreement across domains with near-perfect agreement for AC, MR, UE, OC and PH. Agreement for AC was moderate, indicating variability in expert judgement of the models´s clinical assessment. Copilot demonstrated substantial agreement for AC and PH and moderate agreement across remaining domains. ChatGPT showed moderate agreement for CA and MR but only fair agreement for AC, UE and OC. Agreement for PH was substantial.

In postoperative cases, interrater agreement decreased across all models and domains. For ChatGPT, agreement was low across most domains, with very low agreement for AC. Agreement for PH remained high. For Gemini, agreement was moderate for CA, MR, AC and OC, but poor for UE. Agreement for PH remained high. For Copilot, agreement was moderate across most domains, including UE and OC and substantial for CA. Agreement for PH was moderate. Across models, agreement was highest for CA and PH and the lowest for explanatory domains and confidence ratings, particularly in postoperative scenarios. (Summary of findings in Table 7)

**Table 7:** summary of model performance across evaluation domains.

Model	Strengths	Limitations	Best fit in this dataset
Model A (ChatGPT)	High CA in intraoperative and postoperative cases. High agreement for pH classification	Lower interrater agreement in postoperative cases across several domains. Less separation in postoperative management and UE domains.	Structured recognition tasks under senior clinical oversight.
Model B (Gemini)	Highest postoperative ratings for MR and UE. Highest interrater agreement in intraoperative cases.	Lower interrater agreement for postoperative UE despite high mean ratings.	Postoperative decision support focused on action planning and structured explanations.
Model C (Copilot)	High CA across scenario types. Moderate to substantial agreement across several domains.	Lower intraoperative usefulness vs Gemini. Lower postoperative OC ratings.	Core recognition tasks with caution in explanatory and confidence related domains.

## Discussion

This study evaluated three LLMs in a controlled set of simulated free flap compromise scenarios in head and neck reconstruction. The main finding was that all models received high ratings in recognition-oriented domains, especially clinical accuracy, while greater separation emerged in domains more closely related to management recommendation, explanatory clarity and confidence. These differences were most evident in postoperative scenarios. This pattern is important, but should be interpreted within the limits of the study design. The models were assessed using static, standardized and relatively clean case descriptions. In real clinical practice, flap compromise is a dynamic process. Findings develop over time, information is often incomplete and decisions are made under pressure. Serial changes in flap color, turgor, capillary refill, Doppler signals and systemic status often drive escalation. By contrast, the present scenarios were fixed snapshots. This enabled controlled comparison but also created an experiemental setting likely to favor model performance compared with real-world clinical decision-making.

The finding of high ratings for clinical accuracy should therefore not be interpreted as evidence of clinical readiness. In flap compromise, identifying a likely problem is only one part of the task. The more demanding challenge lies in prioritizing the next step, communicating urgency and escalating appropriately within a narrow salvage window. This aligns with the clinical reality of flap salvage where outcomes depend on rapid recognition, correct interpretation of signs and within a narrow window.^19^^,^^20^ The postoperative findings deserve particular caution. Gemini received highest ratings for management recommendations and usefulness of explanation in postoperative scenarios, while all models retained high ratings for clinical accuracy. This suggests that in static scenario-based testing, the models differed more in how they organized and communicated action than how they labelled the problem itself. However, the study does not show that one model would improve flap salvage, outperform clinicians or function safely in real clinical workflows. The absolute number of responses classified as potentially harmful was low. These events were uncommon, and the finding should be interpreted cautiously given the simulated design and limited number of cases. Potential for harm should therefore be viewed as a safety signal in this simulated dataset, rather than as a stable estimate of clinical risk.

Interrater agreement was higher in intraoperative than postoperative cases across models and domains. This likely reflects the greater uncertainty of postoperative deterioration where expert judgement may vary more in relation to urgency, acceptable thresholds for re-exploration and interpretation of incomplete information. The small expert panel should also be considered when interpreting these findings, since individual rating patterns had greater influence on aggregated scores than in a larger panel. Several rating domains, particularly clinical accuracy and assessment correctness, showed high mean scores across models. This suggests a ceiling effect in the 5-point Likert format, with limited room to discriminate between models at the upper end of performance. As a result, smaller but potentially meaningful differences may have been harder to detect in these domains. A further consideration is the rapid development of commercial LLMs. The present findings reflect the tested model versions and platform interfaces during the study period. They should therefore be interpreted as a time-specific evaluation rather than a fixed estimate of model performance. The same case scenarios repeated after later model updates may produce different diagnostic suggestions, management recommendations, explanations, and expert ratings.

Taken together, the present findings support a narrow and cautious interpretation. In standardized simulated microsurgical scenarios, LLMs showed substantial alignment with expert judgement for core recognition of flap compromise. Their responses differed more when the task required management prioritization, structured escalation and explanatory clarity. This suggests a possible role in education, structured reasoning or supervised support for junior clinicians. It does not support independent clinical use. Prospective evaluation in real clinical workflows using evolving cases and patient-level outcomes is needed before stronger clinical claims can be made.

### Limitations

This study has several limitations. It was based on simulated cases rather than real patient encounters and the scenarios were static, standardized and intentionally structured. This enabled controlled comparison across models and avoided patient risk, but it does not reproduce the evolving and uncertain nature of real flap compromise. In clinical practice, flap deterioration develops over time, often with incomplete information, changing findings and time pressure. The present design therefore evaluates alignment with expert reasoning in standardized snapshots rather than real-time clinical performance. No patient-level outcomes were measured and the study did not assess flap salvage, time to re-exploration, complication rates or morbidity. The findings therefor reflects expert rating of response quality rather than objective clinical effectiveness. The expert panel consisted of four consultant microsurgeons. Although all were experienced, a small panel limits generalizability and increases the influence of individual scoring patterns on collected results, particularly in more subjective domains such as usefulness of explanation and overall confidence. In addition, the use of 5-point Likert scales may have limited discrimination between models in domains with high overall ratings. Ceiling effects were evident for clinical accuracy and assessment correctness, which may have reduced sensitivity for detecting smaller differences at the upper end of performance. LLM output depends on model version, platform interface, system settings, and prompting. The findings therefore represent a time-specific evaluation of the tested systems during the study period and should not be assumed to remain stable after later model updates.

## Conclusion

In standardized simulated scenarios of free flap compromise after head and neck reconstruction, three large language models achieved high expert-rated performance in core recognition tasks. Differences between models became more apparent when responses required management prioritization, explanatory clarity and confidence, particularly in postoperative cases.

These findings suggest a possible role for large language models as structured reasoning or educational support tools in microsurgical settings, especially for junior clinicians working under senior supervision. However, the present study does not establish clinical effectiveness and the static simulated design does not reflect the dynamic and time-sensitive nature of real flap compromise. Large language models should therefore not be used as independent decision-makers in free flap surgery. Prospective studies in real clinical workflows are needed before broader clinical application can be considered.

## Ethics statement

This study used simulated case scenarios and did not involve real patients, patient records, human tissue, or identifiable personal data. Formal ethics committee approval was therefore not required under local institutional regulations.

## Informed consent

No real patient data, images, or identifiable personal information were used in this study. Informed consent was therefore not required.

## Data availability

The data generated and analyzed during this study are available from the corresponding author on reasonable request.

## Use of artificial intelligence

No generative AI was used in writing this manuscript. The three large language models (ChatGPT, Gemin and Copilot) were solely used to generate study data by providing responses to the questions. All text, analysis and interpretation were produced and verified entirely by the authors.

## Funding

This research did not receive any specific grant from funding agencies in the public, commercial, or not-for-profit sectors.

## Meeting presentation

This study will be presented in part at EFSM, Prague, 2026.

## Declaration of competing interest

The authors declare that they have no known competing financial interests or personal relationships that could have appeared to influence the work reported in this paper.

## Appendix Supplementary materials

Image, application 1

Image, application 2

## References

[1] Novakovic D., Patel R.S., Goldstein D.P., Gullane P.J. (2009). Salvage of failed free flaps used in head and neck reconstruction. Head Neck Oncol.

[2] Wolff K., Hölzle F., Wysluch A., Mücke T., Kesting M. (2008). Incidence and time of intraoperative vascular complications in head and neck microsurgery. Microsurgery.

[3] Chen K.T., Mardini S., Chuang D.C.C. (2007). Timing of presentation of the first signs of vascular compromise dictates the salvage outcome of free flap transfers. Plast Reconstr Surg.

[4] Kubo T., Yano K., Hosokawa K. (2002). Management of flaps with compromised venous outflow in head and neck microsurgical reconstruction. Microsurgery.

[5] Kroll S.S., Schusterman M.A., Reece G.P. (1996). Timing of pedicle thrombosis and flap loss after free-tissue transfer. Plast Reconstr Surg.

[6] Hidalgo D.A., Disa J.J., Cordeiro P.G., Hu Q.Y. (1998). A review of 716 consecutive free flaps for oncologic surgical defects: refinement in donor-site selection and technique. Plast Reconstr Surg.

[7] Kamali A., Docherty Skogh A.C., Edsander Nord Å. (2021). Increased salvage rates with early reexploration: a retrospective analysis of 547 free flap cases. J Plast Reconstr Aesthet Surg.

[8] Kiew C.Y.K., Shah A., Hadjiandreou M., Pafitanis G. (2025). Artificial intelligence in microsurgery and supermicrosurgery training within plastic surgery: a systematic review. JPRAS Open.

[9] Song J.K., Rosellini I., Ka Fai W., Yi K.H (2026). Artificial intelligence in plastic surgery and anatomical education: a new era of precision, personalization, and accessibility. J Craniofac Surg.

[10] Gomez-Cabello C.A., Borna S., Pressman S.M., Haider S.A., Forte A.J. (2024). Large language models for intraoperative decision support in plastic surgery: a comparison between ChatGPT-4 and Gemini. Med Kaunas Lith.

[11] Leypold T., Schäfer B., Boos A., Beier J.P. (2023). Can AI think like a plastic surgeon? Evaluating GPT-4’s clinical judgment in reconstructive procedures of the upper extremity. Plast Reconstr Surg Glob Open.

[12] Wang A., Kim E., Oleru O., Seyidova N., Taub P.J. (2024). Artificial intelligence in plastic surgery: chatGPT as a tool to address disparities in health literacy. Plast Reconstr Surg.

[13] Seth I., Cox A., Xie Y. (2023). Evaluating chatbot efficacy for answering frequently asked questions in plastic surgery: a ChatGPT case study focused on breast augmentation. Aesthet Surg J.

[14] Seth I., Bulloch G., Rozen W.M. (2023). Applications of artificial intelligence and large language models to plastic surgery research. Aesthet Surg J.

[15] Zhang A., Li C.X.R., Piper M., Rose J., Chen K., Lin A.Y. (2024). ChatGPT for improving postoperative instructions in multiple fields of plastic surgery. J Plast Reconstr Aesthet Surg JPRAS.

[16] Atkinson C.J., Seth I., Xie Y. (2024). Artificial intelligence language model performance for rapid intraoperative queries in plastic surgery: chatGPT and the deep inferior epigastric perforator flap. J Clin Med.

[17] Holm S., Zambrana M., Berner J.E. (2025). Evaluating artificial intelligence’s role in developing research questions in head and neck reconstruction. Plast Reconstr Surg Glob Open.

[18] Mohapatra D.P., Thiruvoth F.M., Tripathy S. (2023). Leveraging large language models (LLM) for the plastic surgery resident training: do they have a role?. Indian J Plast Surg.

[19] Smit J.M., Acosta R., Zeebregts C.J., Liss A.G., Anniko M., Hartman E.H.M. (2007). Early reintervention of compromised free flaps improves success rate. Microsurgery.

[20] Bui D.T., Cordeiro P.G., Hu Q.Y., Disa J.J., Pusic A., Mehrara B.J. (2007). Free flap reexploration: indications, treatment, and outcomes in 1193 free flaps. Plast Reconstr Surg.

