# Supplementary material for: Artificial intelligence-based decision support in simulated free flap Re-exploration for head and neck reconstruction. A case-based comparative study
Source: JPRAS Open. 2026 May 16;50:606–16. doi: 10.1016/j.jpra.2026.05.010 (PMC13280260; doi:10.1016/j.jpra.2026.05.010)
Supplement: Supplementary file 1 [file mmc1.docx]

# Supplementary table 1

| Intraoperative cases Case 1  History: 59-year-old male, oral squamous cell carcinoma (SCC) of the lower jaw, undergoing fibula free flap reconstruction (FFF).  Intraoperative details: During flap inset, FFF becomes progressively congested with dark purple-blue discoloration.  Examination findings: Slow capillary refill, enlarged veins.  Investigations: Intraoperative doppler shows strong arterial inflow, poor venous outflow sounds.  Photo description: Flap appears dark purple with visible venous enlargements. |
| --- |
| Case 2  History: 67-year-old female, tongue reconstruction post-glossectomy, free radial forearm flap  Intraoperative details: After completing arterial anastomosis, flap starts to appear pale.  Examination findings: Delay of capillary refill, no bleeding from skin edges on the free flap.  Investigations: No intraoperative doppler signal at the arterial anastomosis.  Photo description: Pale, white ischemic flap surface with no visible bleeding. |
| Case 3  History: 49-year-old woman, buccal reconstruction due to squamous cell carcinoma with a free radial forearm flap  Intraoperative details: Venous congestion appears after inset of the free radial forearm flap.  Examination findings: Flap becomes rapidly bluish-purple and turgid.  Investigations: Exploration reveals the vein kinking.  Photo description: Purple discoloration partly over the flap (50%). |
| Case 4  History: 70-year-old male, scalp reconstruction due to extensive basal cell carcinoma, with an ALT flap.  Intraoperative details: Abrupt loss of intraoperative doppler from the arterial anastomosis after clamp removal.  Examination findings: Flap turns cold and pale.  Investigations: Exploration reveals thrombosis in arterial anastomosis.  Photo description: White flap compared to the rest of the scalp. |
| Case 5  History: 61-year-old female, oromandibular reconstruction with a free radial forearm flap.  Intraoperative details: Flap initially pink, then rapidly congested turning purple.  Examination findings: Slow capillary refill and engorged vein proximal to anastomosis.  Investigations: On revision during operation, venous clot found at anastomosis site.  Photo description: Dark and purple congested flap with engorged vein is visible in the mouth. |
| Case 6  History: 45-year-old female, ALT flap for tongue defect due to squamous cell carcinoma.  Intraoperative details: Operation temperature drops during the reconstruction.  Examination findings: Flap pale, capillary refill is absent.  Investigations: No arterial intraoperative doppler signal, improved with warming.  Photo description: Pale flap with shrunken vessels, purple discoloration of one edge of the flap in the mouth. |
| Case 7  History: 54-year-old male, free fibula flap for mandible after trauma  Intraoperative details: After inset of the flap, the arterial signal lost with intraoperative doppler.  Examination findings: Flap cool and pale. No existing intraoperative doppler sound.  Investigations: Exploration reveals kink in artery due to positioning of the pedicle.  Photo description: Flap with patchy pallor, partly pale. |
| Case 8  History: 65-year-old male, oral cancer and bone resection. Reconstruction with a free scapula flap.  Intraoperative details: Flap arterial flow compromised after anastomosis  Examination findings: Weak arterial intraoperative doppler sounds, delayed capillary refill.  Investigations: Excessive vessel tension is found during exploration.  Photo description: Slightly pale flap is seen. |
| Case 9  History: 50-year-old female, ALT flap to the neck after a full-thickness burn.  Intraoperative details: Drop in blood pressure during anaesthesia.  Examination findings: Flap pale but warmer and more pink with blood pressure correction.  Investigations: Arterial intraoperative doppler is present but weaker during hypotension.  Photo description: Transient pallor on the ALT flap. |
| Case 10  History: 57-year-old male, free radial forearm flap to the ear after cancer resection.  Intraoperative details: Flap is congested rapidly before closure.  Examination findings: Engorged veins, firm swelling under skin which is getting larger.  Investigations: Hematoma is compressing the venous pedicle.  Photo description: Purple flap with a tense swelling at pedicle site. |
| Case 11  History: 75-year-old woman, scalp defect reconstruction due to malignant melanoma, reconstructed with a free latissimus dorsi flap.  Intraoperative details: Artery inflow is slower despite patent anastomosis.  Examination findings: Patchy discolouration of the flap.  Investigations: Backflow test slow.  Photo description: Irregular and patchy pink flap. |
| Case 12  History: 55-year-old woman, large cheek defect due to basal cell carcinoma. Reconstructed with an ALT flap.  Intraoperative details: Distal areas of the flap remain cyanotic.  Examination findings: Different color from proximal pink to distal purple.  Investigations: Intraoperative doppler is strong proximally and weak distally.  Photo description: A distal flap congestion is seen. |
| Case 13  History: 62-year-old male, mandibular reconstruction with a free fibula flap due to squamos cell carcinoma.  Intraoperative details: Venous congestion is seen after final inset.  Examination findings: Swollen flap, poor intraoperative doppler venous signal.  Investigations: Pedicle twist can be seen on exploration.  Photo description: Engorged, purple and tense flap. |
| Case 14  History: 47-year-old woman, scalp reconstruction with an ALT flap after squamous cell carcinoma.  Intraoperative details: Arterial wall injury during dissection of the recipient vessels.  Examination findings: Poor perfusion is seen after anastomosis.  Investigations: intraoperative doppler sounds is absent distal to the vessel injury.  Photo description: Ischemic and pale flap. |
| Case 15  History: 59-year-old female, tongue reconstruction with a free radial flap due to cancer.  Intraoperative details: Venous congestion is seen after tunnelling the pedicle.  Examination findings: Swollen and a purple flap.  Investigations: Release of the tunnel improves the flow.  Photo description: Purple and congested flap. |

# Postoperative Cases

| Case 1  History: 60-year-old male, fibula flap due to mandibular reconstruction.  Postoperative details: Flap darkens in colour compared to directly postoperative at ICU.  Examination findings: Slow capillary refill and venous doppler absent.  Investigations: No venous doppler sounds  Photo description: Dark purple flap. |
| --- |
| Case 2  History: 67-year-old female, ALT flap reconstruction due to tongue cancer.  Postoperative details: Flap becomes cold and pale postoperatively on the ward.  Examination findings: No bleeding on pinprick with needle.  Investigations: No arterial Doppler is found.  Photo description: White and ischemic flap. |
| Case 3  History: 59-year-old male, free radial forearm flap to buccal mucosa due to squamous cell carcinoma.  Postoperative details: Increasing swelling and darkening color on the ward.  Examination findings: Tense flap with sluggish refill.  Investigations: Doppler sound shows arterial signal intact.  Photo description: Congested and swollen flap. |
| Case 4  History: 68-year-old female, reconstructed with a free fibula flap due to cancer and mandibular reconstruction.  Postoperative details: Sudden pallor is seen.  Examination findings: Cool flap with no Doppler signal on the ward.  Investigations: No arterial signal is heard on handheld Doppler.  Photo description: Ischemic and white flap. |
| Case 5  History: 66-year-old male, scalp reconstruction due to squamous cell carcinoma, with an ALT flap.  Postoperative details: Swelling is seen at pedicle site.  Examination findings: Venous congestion signs with purple colour.  Investigations: Ultrasound at the ward shows hematoma.  Photo description: Purple flap with a localized swelling. |
| Case 6  History: 75-year-old male, free radial forearm flap to floor of the mouth due to cancer.  Postoperative details: Blood pressure drop overnight at the ward.  Examination findings: Flap starts to be pale and warms after blood pressure correction.  Investigations: Weak but present Doppler sound during hypotension.  Photo description: Pallor which is resolved post-resuscitation. |
| Case 7  History: 60-year-old female, ALT flap reconstruction due to cancer of the cheek.  Postoperative details: Dressing is too tight.  Examination findings: Venous congestion is seen with purple colour of the flap.  Investigations: Immediate improvement is seen after dressing release.  Photo description: Purple flap is returning to pink colour. |
| Case 8  History: 69-year-old male, free fibula flap reconstruction due to trauma.  Postoperative details: There is a cold environment in ICU.  Examination findings: Pale and white flap.  Investigations: Doppler signals are weak which improves with warming.  Photo description: Cool and pale flap. |
| Case 9  History: 67-year-old male, scalp reconstruction with a large latissimus dorsi flap due to cancer.  Postoperative details: Erythema and swelling is seen.  Examination findings: Venous congestion signs with purple flap.  Investigations: CRP elevated, fever and Doppler signals are normal.  Photo description: Red and congested flap. |
| Case 10  History: 55-year-old female, free radial forearm flap to the oral cavity due to cancer.  Postoperative details: Sudden flap congestion is seen at the ward after patient movement. Early mobilisation of the neck.  Examination findings: Tense and purple flap.  Investigations: No Doppler signals is heard.  Photo description: Dark purple flap with swelling is seen. |
| Case 11  History: 59-year-old male, cheek reconstruction with a free radial forearm flap due to basal cell carcinoma.  Postoperative details: Distal area turns black over days at the ward.  Examination findings: Demarcated necrosis of the superior part of the flap and the proximal flap is healthy.  Investigations: Distal perfusion is poor, no bleeding.  Photo description: Black distal segment on the flap. |
| Case 12  History: 56-year-old female, free radial forearm flap to the occipital area due to trauma.  Postoperative details: Gradual is swelling is seen on the ward.  Examination findings: Slow capillary refill with venous Doppler signals being absent.  Investigations: Swelling at the site of the flap.  Photo description: Purple and tense flap.  Case 13  History: 68-year-old male, late postoperative day 7 after a neck reconstruction with a free ALT flap due to full-thickness burn.  Postoperative details: Progressive pallor is seen.  Examination findings: Weak Doppler signal, especially arterial sounds.  Investigations: No signs of infections. More difficult with mobilization of the neck.  Photo description: Pale and cool flap. |
| Case 14  History: 52-year-old female, free ALT flap reconstruction to the oral cavity due to cancer.  Postoperative details: Swelling is seen near pedicle.  Examination findings: Venous congestion and purple colour.  Investigations: Ultrasound shows fluid collection near the pedicle.  Photo description: Purple colour of the flap with swelling. |
| Case 15  History: 60-year-old male, free fibula flap reconstruction after a gun wound to the jaw.  Postoperative details: Flap discoloration with purple colour at the ward.  Examination findings: Venous Doppler absent but the arterial signals are intact.  Investigations: No signs of infection. No swelling.  Photo description: Dark purple congested flap |
