# Supplementary material for: Artificial intelligence-based decision support in simulated free flap Re-exploration for head and neck reconstruction. A case-based comparative study
Source: JPRAS Open. 2026 May 16;50:606–16. doi: 10.1016/j.jpra.2026.05.010 (PMC13280260; doi:10.1016/j.jpra.2026.05.010)
Supplement: Supplementary file 2 [file mmc2.docx]

# Prompt, Supplementary table 2

| You are assisting a plastic surgeon managing a free flap reconstruction in the head and neck. Based on the following clinical scenario, please provide: |
| --- |
| 1. Your suggested diagnosis. |
| 2. A clear action plan. |
| 3. Recommendations for further management, including any investigations or escalation. |
